# *agr*-Mediated Dispersal of *Staphylococcus aureus* Biofilms

**DOI:** 10.1371/journal.ppat.1000052

**Published:** 2008-04-25

**Authors:** Blaise R. Boles, Alexander R. Horswill

**Affiliations:** Department of Microbiology, Roy J. and Lucille A. Carver College of Medicine, University of Iowa, Iowa City, Iowa, United States of America; Institut Pasteur, France

## Abstract

The *agr* quorum-sensing system of *Staphylococcus aureus* modulates the expression of virulence factors in response to autoinducing peptides (AIPs). Recent studies have suggested a role for the *agr* system in *S. aureus* biofilm development, as *agr* mutants exhibit a high propensity to form biofilms, and cells dispersing from a biofilm have been observed displaying an active *agr* system. Here, we report that repression of *agr* is necessary to form a biofilm and that reactivation of *agr* in established biofilms through AIP addition or glucose depletion triggers detachment. Inhibitory AIP molecules did not induce detachment and an *agr* mutant was non-responsive, indicating a dependence on a functional, active *agr* system for dispersal. Biofilm detachment occurred in multiple *S. aureus* strains possessing divergent *agr* systems, suggesting it is a general *S. aureus* phenomenon. Importantly, detachment also restored sensitivity of the dispersed cells to the antibiotic rifampicin. Proteinase K inhibited biofilm formation and dispersed established biofilms, suggesting *agr*-mediated detachment occurred in an *ica*-independent manner. Consistent with a protease-mediated mechanism, increased levels of serine proteases were detected in detaching biofilm effluents, and the serine protease inhibitor PMSF reduced the degree of *agr*-mediated detachment. Through genetic analysis, a double mutant in the *agr*-regulated Aur metalloprotease and the SplABCDEF serine proteases displayed minimal extracellular protease activity, improved biofilm formation, and a strongly attenuated detachment phenotype. These findings indicate that induction of the *agr* system in established *S. aureus* biofilms detaches cells and demonstrate that the dispersal mechanism requires extracellular protease activity.

## Author Summary

A biofilm is a surface-attached community of cells bound together by an extracellular matrix. In a bacterial infection, biofilm-encased cells are protected from antibiotic therapy and host immune response, and these encased cells can develop into a chronic infection. *Staphylococcus aureus* is a prominent bacterial pathogen known to form biofilms on many medical implants and host tissues. In this report, we demonstrate that repression of the *S. aureus* quorum-sensing system is required to form a biofilm, and quorum-sensing reactivation in established biofilms disperses the cells. Genetic and molecular analysis demonstrates that quorum-sensing is activated before and required for the detachment mechanism. Detachment is protease-mediated, as established biofilms are sensitive to a non-specific protease and quorum-sensing activation increases the production of extracellular proteases. Using mutations in the protease genes, we show that these secreted enzymes are required for the detachment mechanism. These findings denote that *S. aureus* quorum-sensing can function as a dispersal mechanism to colonize new sites, and our results suggest this mechanism could be modulated to treat recalcitrant biofilms.

## Introduction

Most bacteria have an inherent ability to form surface-attached communities of cells called biofilms [1]. The opportunistic pathogen *Staphylococcus aureus* can form biofilms on many host tissues and implanted medical devices often causing chronic infections [2]–[5]. The challenge presented by biofilm infections is the remarkable resistance to both host immune responses and available chemotherapies [6],[7], and estimates suggest that as many as 80% of chronic bacterial infections are biofilm associated [8]. In response to certain environmental cues, bacteria living in biofilms are capable of using active mechanisms to leave biofilms and return to the planktonic (free-living) state in which sensitivity to antimicrobials is regained [9]–[11]. Therefore an improved understanding of the molecular mechanism of biofilm detachment could facilitate the discovery of innovative treatment options.

Studies on the opportunistic pathogen *Pseudomonas aeruginosa* indicate that cell-to-cell communication (often termed “quorum-sensing”) is required to make a robust biofilm under some growth conditions [12]. Surprisingly, the opposite is true in *S. aureus*, as the presence of an active quorum-sensing impedes attachment and development of a biofilm [13],[14]. The *S. aureus* quorum-sensing system is encoded by the accessory gene regulator (*agr*) locus and the communication molecule that it produces and senses is called an autoinducing peptide (AIP), which is an eight-residue peptide with the last five residues constrained in a cyclic thiolactone ring [15]. During growth, AIP is synthesized and secreted through a poorly understood mechanism that requires multiple peptidases [16],[17]. Once AIP reaches a critical concentration, it binds to a surface histidine kinase receptor, initiating a regulatory cascade that controls expression of a myriad of virulence factors, such as proteases, hemolysins, and toxins [18]. A recent study by Yarwood *et. al.* [19] raised the possibility that the *agr* quorum-sensing system is involved in biofilm detachment. This study demonstrated that bacteria dispersing from biofilms displayed high levels of *agr* activity, while cells in a biofilm had predominantly repressed *agr* systems. These findings correlate well with prior data indicating that *agr* deficient *S. aureus* strains form more robust biofilms compared to wild type strains [13],[14].

In the study presented here, we demonstrate that activation of the *agr* system in established biofilms is necessary for detachment. This activation could be accomplished with exogenous AIP addition or by changing nutrient availability to the biofilm. We also demonstrate that *agr*-mediated detachment requires the activity of extracellular proteases. Our findings suggest that *agr* quorum-sensing is an important regulatory switch between planktonic and biofilm lifestyles and may contribute to *S. aureus* dispersal and colonization of new sites.

## Results

### Low *agr* activity is important for biofilm development

**Figure 1:**
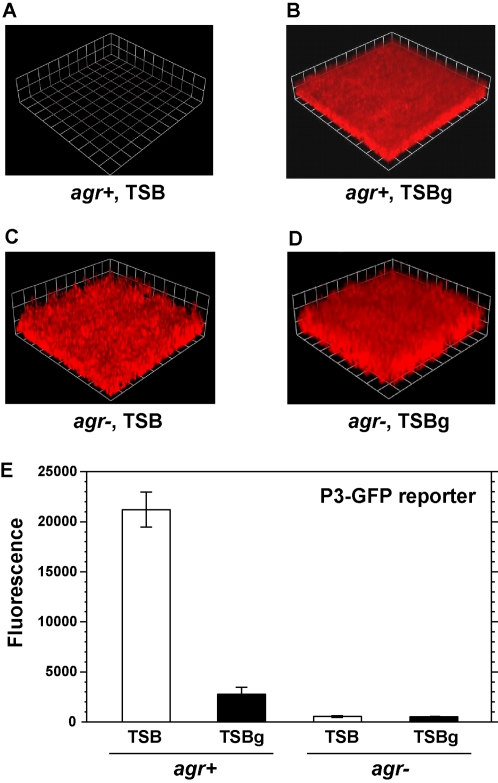
Low *agr* activity is important for *S. aureus* biofilm formation. (A–D) Biofilms were grown for 2 days in either 2% TSB or 2% TSB supplemented with 0.2% glucose (referred to as “TSBg”). Biofilm integrity and RFP fluorescence were monitored with CLSM. Three dimensional image reconstructions of a *z* series were created with Velocity software. CLSM images are representative of three separate experiments and each side of a grid square represents 20 µM. (A) AH596 (*agr*+) grown in TSB. (B) AH596 grown in TSBg. (C) AH871 (*agr*-) grown in TSB. (D) AH871 grown in TSBg. (E) Measurement of the *agr* P_3_-GFP reporter (pDB59) activity in strains AH596 and AH871 grown in broth culture in either TSB or TSBg. Error bars show standard error of the mean (SEM).

Mutations in the *agr* quorum-sensing system are known to improve biofilm development [13],[14]. Based on these studies, it seemed probable that there is a correlation between *agr* activity and biofilm formation. Regassa et al. reported that growth on rich media containing glucose represses the *agr* system through the nonmaintained generation of low pH [20]. Interestingly, in most published flow cell biofilm studies, one commonality is the use of growth media containing or supplemented with glucose [9], [19], [21]–[24]. In our own efforts to grow *S. aureus* flow cell biofilms, we found a strict dependence on glucose supplementation. For the experimental setup, a once-through, continuous culture system was employed as previously described [19],[25], and *S. aureus* SH1000 constitutively expressing red fluorescent protein (P*_sarA_*-RFP, plasmid pAH9) was used as the testing strain. Using 2% TSB as the growth media, SH1000 cells did not attach and develop a biofilm (Figure 1A), instead passing right through the flow cell to the effluent. However, in the presence of 0.2% glucose (TSBg), cells attached and a formed a robust biofilm (10–20 microns thick) after two days of growth, which was visually evident and monitored with confocal laser scanning microscopy (CLSM, Figure 1B). As expected, glucose strongly inhibited expression from the P3 promoter using a GFP reporter (Figure 1E), suggesting that repression of RNAIII is essential for attachment and biofilm formation. In broth culture and biofilm effluents, we observed a glucose-dependent pH decrease to the 5.5 range similar as previously reported [20],[26]. As a control, flow cell biofilms were prepared with an *agr* mutant strain (SH1001, *Δagr*::TetM) containing plasmid pAH9 (Figure 1C & D), and this strain developed a biofilm even in the absence of media supplementations (Figure 1C). As anticipated, the P3 promoter did not activate in the *agr* mutant (Figure 1E). Overall, these observations indicate that environmental conditions favoring low *agr* activity are essential for attachment and biofilm formation.

### AIP detaches *S. aureus* biofilms

**Figure 2:**
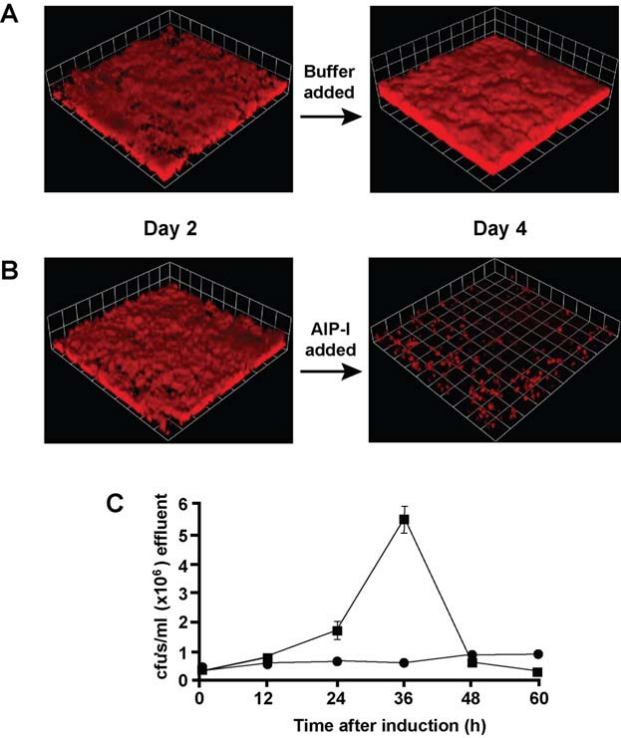
Detachment of *S. aureus* biofilms with AIP. Biofilms (strain AH500) were grown in flow cells for 2 days. Either (A) 1 mL of buffer (100 mM phosphate [pH 7], 50 mM NaCl, 1 mM TCEP) or (B) 1 mL of 20 µM AIP-I in buffer was diluted 1000-fold into the biofilm growth media. The biofilm integrity was monitored with CLSM for 2 more days. Each side of a grid square in the image reconstructions represents 20 µM. (C) Effect of AIP-I addition on number of detached bacteria in the effluent medium from flow cell biofilms. The plot depicts CFU/ml in effluents from biofilms, and the black squares (▪) represent AIP-I addition and the black circles (•) represent buffer addition to the biofilm. Graph shows the mean of 3 effluent collections from 1 experiment, error bars show SEM.

To investigate the role of the *agr* system in established biofilms, we developed strategies to modulate level of *agr* activity within a biofilm. Initially, media supplementation experiments were performed using purified AIP signal in order to place the *agr* system under external control. We recently developed a new method for AIP biosynthesis [27], enabling the production of sufficient signal levels for flow cell experiments. Through exogenous AIP addition, we could test wild-type strains and avoid any potential complications of constructed *agr* deletion mutants. For this approach, established flow cell biofilms were prepared using *S. aureus* SH1000 constitutively expressing RFP with plasmid pAH9. The flow cell media was supplemented with glucose to attenuate *agr* expression [20], allowing cell attachment and biofilm development. After two days, either 1 mL of buffer (100 mM phosphate [pH 7], 50 mM NaCl, 1 mM TCEP; Figure 2A) or 1 mL of 20 µM AIP-I in buffer (Figure 2B and Video S1) was diluted 1000-fold (50 nM final concentration) into the growth media. Using our synthesized AIP-I in dose-response curves [27], we estimate the amount of AIP-I in supernatants of TSB broth cultures (OD_600_ 1.0–1.3) reaches approximately 400 nM (data not shown), indicating the 50 nM level used for the biofilm experiments is within a relevant concentration range. Examination with CLSM showed that the AIP-I treated biofilm sloughed off the flow cell over a period of 1–2 days (Figure 2B and Video S1), suggesting that AIP-I activated a detachment mechanism. To confirm that AIP-I caused detachment, we counted viable *S. aureus* cells in the effluent media (Figure 2C). The concentration of bacteria in the effluent increased markedly 24–36 hours after AIP-I addition. In contrast, the number of bacteria in the biofilm effluent without AIP-I addition remained relatively constant. Computational analysis of the detachment phenotype indicated that 91.3±4.3% of the biomass dispersed within 48 hrs of AIP-I addition.

### AIP-mediated biofilm detachment is a general phenomenon

Among *S. aureus* strains, there are four types of *agr* quorum-sensing systems. Each of these *agr* systems, referred to as *agr*-I through *agr*-IV, recognizes a unique AIP structure (AIP-I through AIP-IV). Through an intriguing mechanism of chemical communication, these varying quorum-sensing systems can be subdivided into three cross-inhibitory groups: *agr*-I/IV, *agr*-II, and *agr*-III. The activating signals of each group cross-inhibits the alternative signal receptors with surprising potency, a phenomenon termed “bacterial interference” [15]. Since AIP-I and AIP-IV differ by only one amino acid and function interchangeably [28], they are grouped together in the classification scheme, although this assignment has been controversial [29],[30].

**Figure 3:**
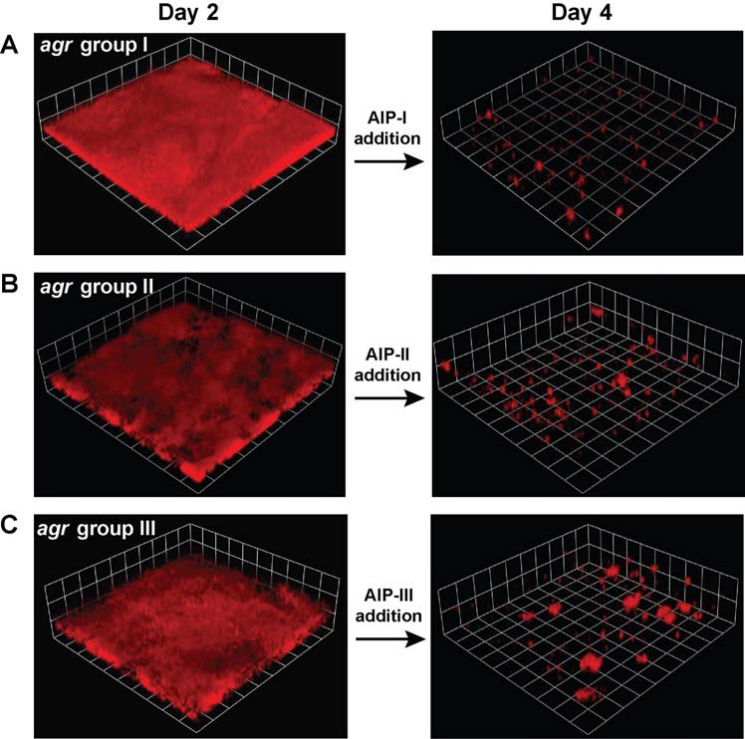
Effect of AIP addition to biofilms from *S. aureus* strains representing different *agr* classes. Biofilms were grown in flow cells for 2 days and indicated AIP was added (50 nM final concentration) to the growth media. Biofilm integrity was monitored with CLSM. Each side of a grid square in the image reconstructions represents 20 µM, and red color is from propidium iodide stain present in growth medium. (A) Biofilm of strain FRI1169 (*agr* Type I) treated with AIP-I. (B) Biofilm of strain SA502A (*agr* Type II) treated with AIP-II. (C) Biofilm of strain ATCC25923 (*agr* Type III) treated with AIP-III.

To determine the generality of the detachment mechanism, we examined the effect of AIP addition using *S. aureus* strains representing different *agr* groups. The strains tested were (i) FRI1169, *agr*-I, toxic shock syndrome isolate [31]; (ii) SA502a (ATCC27217), nasal isolate and prototype *agr*-II strain [15],[32]; and (iii) ATCC25923, clinical *agr*-III isolate [9]. When the correct AIP signal was added to 2-day old biofilms of each strain (FRI1169, AIP-1; SA502a, AIP-II; ATCC25923, AIP-III), signal addition resulted in robust detachment of each biofilm over a period of 48 hours (Figure 3). These findings indicate biofilm detachment is a general *S. aureus* phenomenon that occurs in laboratory strains and clinical isolates, and functions across diverse *agr* systems.

### The timing and requirement of the *agr* system in detachment

**Figure 4:**
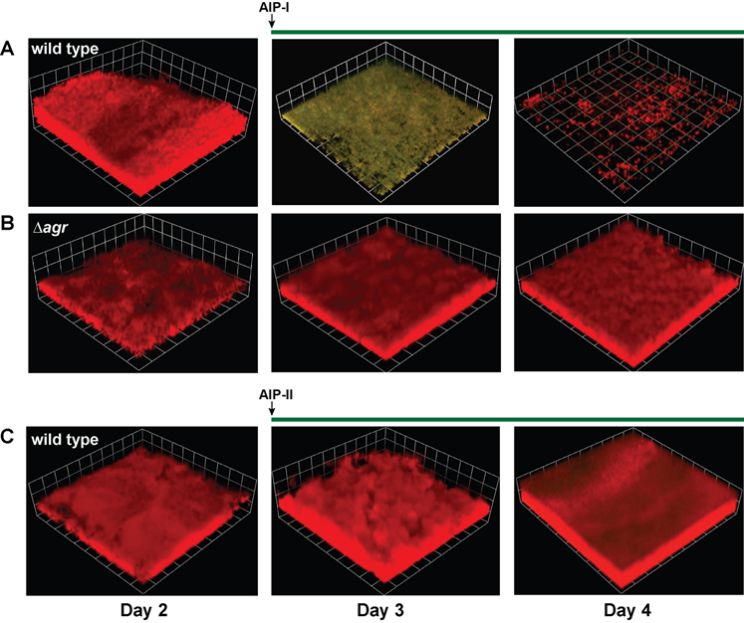
Expression of *agr* P_3_ promoter in biofilms after AIP addition. Dual-labeled biofilms (P*_sarA_*-RFP, P*_agrP3_*-GFP) were grown for 2 days, and AIP-I (50 nM final) was added to the growth media. Biofilm integrity and RFP/GFP fluorescence were monitored with CLSM at day 3 and 4. Greenish yellow color indicates expression of the *agr* P_3_-GFP reporter (pDB59). (A) Addition of AIP-I to an *agr* type I wild type strain (AH596) or (B) *agr* deficient strain (AH861). (C) Addition of interfering AIP-II to an *agr* type-I strain biofilm (AH596). CLSM image reconstructions are representative of three separate experiments and each side of a grid square represents 20 µM.

If AIP was promoting biofilm detachment via the *agr* system, we predicted that *agr* expression would be induced prior to detachment and an *agr* deficient mutant would not detach in response to AIP. To determine whether the *agr* system is activated prior to biofilm detachment, a dual fluorescent-labeled SH1000 strain was constructed with a constitutive RFP (P*_sarA_*-RFP, pAH9) and an *agr* responsive GFP reporter (P*_agrP3_*-GFP, pDB59). After two days of biofilm growth, we added AIP-I to the biofilm flow medium and this resulted in strong induction of the GFP reporter (Figure 4A), indicating activation of the *agr* system. As shown, the GFP reporter was clearly activated before dispersal of the biofilm cells. By the fourth day, all cells with detectable GFP expression detached from the biofilm. These observations provide convincing evidence that AIP activates the *agr* system prior to biofilm dispersal.

To further investigate the role of the *agr* system, we utilized a mutant strain with a complete deletion of the *agr* locus (SH1001). Unlike the wild type strain (Figure 4A), the *agr* mutant biofilm harboring the same dual reporters did not respond to AIP-I treatment, as evidenced by a lack of GFP induction, and the mutant biofilm did not disperse (Figure 4B). Similarly, addition of an inhibitory AIP (50 nM AIP-II) to the dual-labeled SH1000 biofilm failed to induce GFP expression, and again, the biofilm did not disperse (Figure 4C). Taken together, these data demonstrate that an active *agr* quorum-sensing system is necessary for AIP-mediated biofilm dispersal.

### Changing environmental conditions can induce detachment

**Figure 5:**
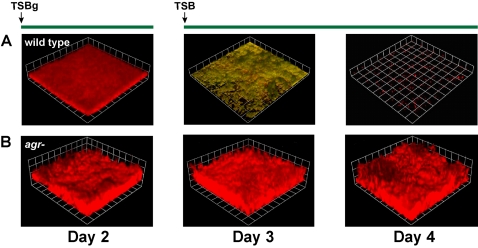
Effect of changing growth conditions on *agr*-mediated biofilm detachment. Dual-labeled biofilms (P*_sarA_*-RFP, P*_agrP3_*-GFP) of (A) *agr* positive strain AH596 and (B) *agr* mutant strain AH871 were grown for 2 days in TSBg. Glucose was removed from the growth media and the biofilm was grown an additional 2 days. Biofilm integrity and RFP/GFP fluorescence were monitored with CLSM. CLSM image reconstructions are representative of three separate experiments and each side of a grid square represents 20 µM.

We have demonstrated that low *agr* activity is important for biofilm formation and that activation of the *agr* system in established biofilms induces detachment. Considering changes to the physiochemical environment may occur *in vivo*, we investigated whether an alteration in nutrient availability could reproduce the detachment phenotype. Again, two day flow cell biofilms were prepared with the dual-labeled strain (AH596) in TSBg (Figure 5A). The glucose was removed and significant activation of the P3 promoter was apparent by monitoring GFP levels using CLSM (Figure 5A), supporting our previous result (Figure 1A). Once the *agr* system was activated, robust detachment from the flow cell was observed and monitored with CLSM (Figure 5A). An *agr* deletion mutant did not respond to glucose depletion (Figure 5B), indicating the detachment phenotype was dependent upon a functional *agr* system. These findings demonstrated that glucose depletion can disperse an *S. aureus* biofilm and again the detachment occurred through an *agr*-dependent mechanism. These experimental observations mirrored those with AIP addition and further support the apparent inverse correlation between *agr* activity and biofilm formation.

### Detached *S. aureus* cells regain antibiotic sensitivity

**Figure 6:**
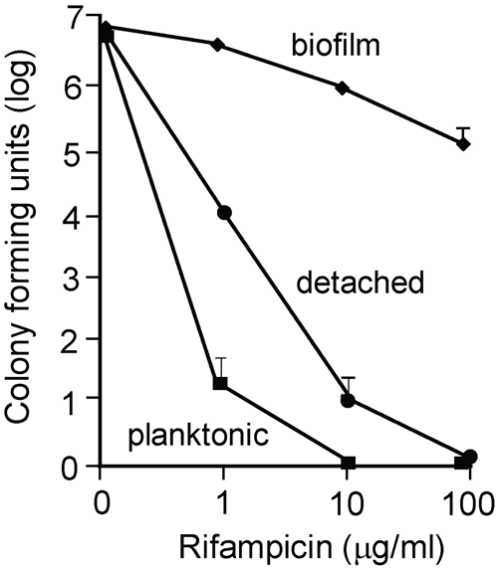
Susceptibility of biofilm and detached bacteria to rifampicin killing. *S. aureus* SH1000 biofilm bacteria (black diamonds) were grown in flow cells containing removable coupons, allowing multiple replicate biofilms to be exposed to rifampicin and surviving CFU's to be determined. Detached bacteria (black circles) were collected from flow cell effluents of biofilms exposed to AIP-I. As a control, planktonic bacteria (black squares) were treated with the same level of rifampicin. Graph show the mean of three experiments; error bars show SEM.

Biofilm growth of *S. aureus* increases resistance to antimicrobials when compared to the planktonic growth mode [9],[19]. This biofilm mediated resistance hinders treatment of many chronic *S. aureus* biofilm related infections, including endocarditis, osteomyelitis, and indwelling medical device infections [3],[33]. Therefore, we asked whether AIP-dispersed bacteria regained sensitivity to a clinically relevant antibiotic, rifampicin. To test this, we collected detached cells from an AIP-treated biofilm effluent and compared resistance to intact biofilms exposed to different levels of rifampicin. Similar to previous antibiotic susceptibility results [19], even at the highest concentration tested (100 µg/ml), the level of rifampicin killing was <2 log units of the biofilm biomass (Figure 6). In contrast, the viability of detached cells displayed a different antibiotic response. At 10 µg/ml rifampicin, a 6 log decrease of viable cells was detected, and at 100 µg/ml, complete killing of the detached cells was observed (Figure 6). The AIP-detached cells were more resistant than broth culture to comparable levels of rifampicin, suggesting parts of the detached biofilm may remain in emboli that are known to possess elevated antibiotic resistance [9]. These observations demonstrated that *S. aureus* cells detached from a biofilm regain susceptibility to a clinical antibiotic.

### The role of PIA in biofilm detachment

**Figure 7:**
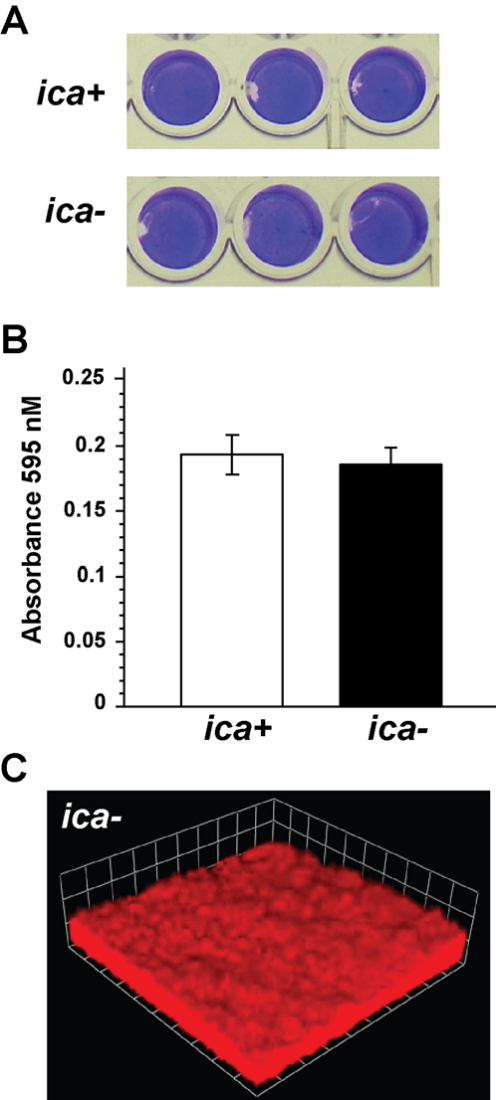
Role of *ica* locus in biofilm development. (A) Microtiter biofilms of *ica* positive strain SH1000 and *ica* deletion mutant AH595. (B) Quantitation of microtiter biofilms. (C) Representative CLSM image of flow cells biofilms of strain AH595 grow in TSBg for 2 days. Each side of a grid square represents 20 µM, and red color is from propidium iodide stain present in growth medium.

*S. aureus* possesses the *icaRADBC* locus that is required to synthesize and generate an exopolysaccharide, which is referred to as the polysaccharide intracellular adhesin or PIA (also called PNAG). *S. aureus* is known to form biofilms through both *ica*-dependent and *ica*-independent mechanisms [34],[35]. To gain insight on the biofilm detachment mechanism, we sought to distinguish whether our *S. aureus* biofilms were dependent on PIA. In strain SH1000, we constructed an Δ*ica*::Tet deletion mutant (strain AH595) using generalized transduction and confirmed the mutation with PCR and sequencing. In microtiter biofilm assays, we were unable to identify a biofilm phenotype (Figure 7A and 7B). Similarly in flow cell biofilms, we did not observe a defect in the ability of strain AH595 to form a biofilm (Figure 7C). No difference was observed compared to flow cell biofilms of SH1000 grown in parallel (data not shown). While SH1000 is a derivative of 8325-4, and there are reports that the *ica* locus is required for 8325-4 derived strains to make a biofilm [36], the *ica* locus was not required for biofilm formation under our experimental conditions. Similar to our observations, an *ica* mutant of the clinical *S. aureus* isolate UAMS-1 displays no defect in microtiter and flow cell biofilm assays [22]. In contrast, when proteinase K was added to SH1000, biofilms were unable to develop in the microtiter plate format (data not shown), indicating the biofilms are forming through an *ica*-independent mechanism. These findings suggest that PIA is unlikely to have a role in biofilm detachment in the SH1000 strain background.

### Investigating the biofilm detachment mechanism

Knowing the *agr* system is essential for biofilm detachment, what *agr* regulated products are responsible for the dispersal phenotype? In *S. aureus* strains that produce *ica*-independent biofilms, proteinase K eliminates adherence and biofilm formation [35], [37]–[39], perhaps through cleavage of surface structures. *S. aureus* is coated with cell wall attached proteins that mediate adherence to a variety of substrates [40], and some of these adhesins, such as biofilm associated protein (BAP) and SasG are important for biofilm formation [41],[42]. It is also known that some surface adhesins, such as protein A and fibronectin-binding protein, are cleaved by the native *S. aureus* secreted proteases [43],[44]. Considering the *agr* system regulates the secreted proteases [45],[46], we hypothesized that increased expression of extracellular proteases could be responsible for biofilm detachment.

**Figure 8:**
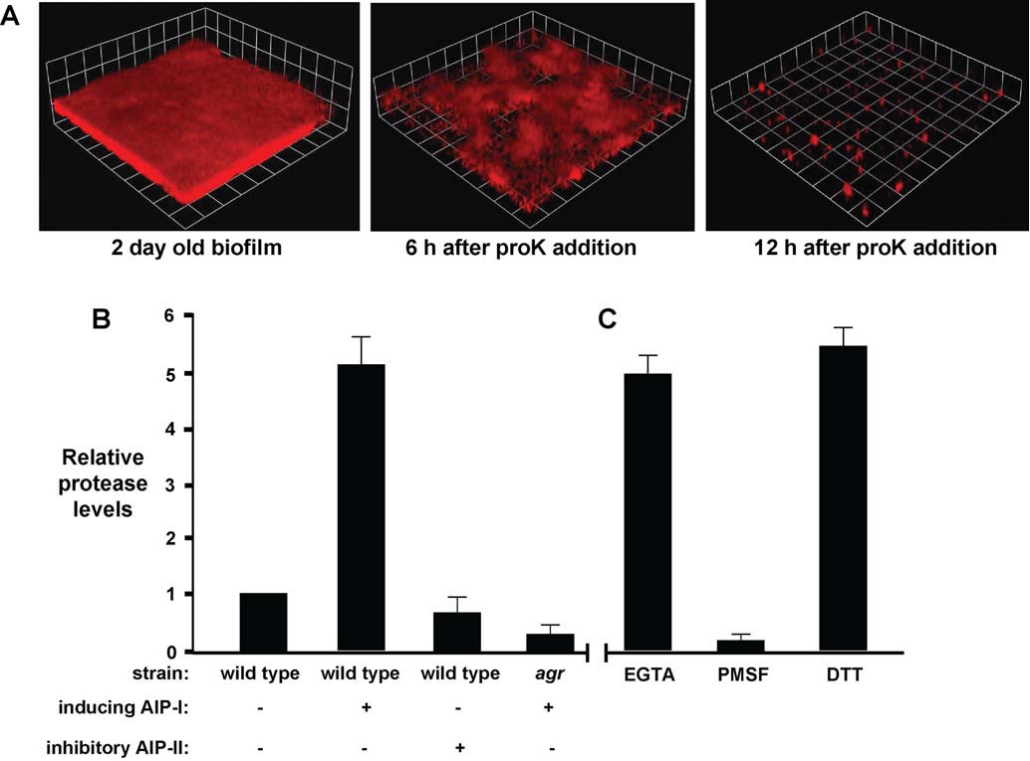
Effect of Proteinase K on biofilms and measurement of extracellular protease activity in AIP-detached biofilms. (A) Proteinase K (proK, 2 µg/ml) was added to a 2 day old biofilm (strain AH500) and the biofilm integrity was monitored with CLSM at 6 and 12 hr. (B) Levels of protease activity detected in biofilm effluent collected from wild type (SH1000) or Δ*agr* (SH1001) biofilms supplemented with indicated AIP's. Protease activity was referenced to wild-type without AIP addition. (C) The effect of inhibitors or activators on the proteolytic activity in an AIP-I detached biofilm effluent. Activity assay was supplemented with either the metalloprotease inhibitor EGTA (1 mM), serine protease inhibitor PMSF (10 µM), or the reducing agent DTT (1 mM). Error bars show SEM.

If *S. aureus* proteases have a role in detachment, proteinase K should be able to disperse an established biofilm. To test this proposal, proteinase K (2 µg/mL) was added to a SH1000 biofilm and resulted in rapid detachment over 12 hrs (Figure 8A). With this preliminary observation, we measured the levels of protease activity in effluents from biofilms with and without AIP-I addition using Azocoll (azo dye impregnated collagen) reagent. As shown in Figure 8B, we detected a baseline level of protease activity in biofilm effluents without AIP-I addition and referenced other measurements to this baseline. With the addition of activating AIP-I, the protease activity increased approximately five-fold compared to a biofilm with no AIP-I treatment. As anticipated, addition of inhibitory AIP-II reduced the level of proteolytic activity in the effluent. Similarly, an *agr* mutant biofilm supplemented with activating AIP-I displayed very low levels of extracellular proteases (Figure 8B).

There are 10 known extracellular proteases produced by most *S. aureus* strains and expression of all these enzymes is controlled by the *agr* system [18],[45],[46]. These 10 proteases include the metalloprotease aureolysin (*aur*), two cysteine proteases (*scpA* and *sspB*), and seven serine proteases (*sspA* (V8) and *splABCDEF*) [47]. To elucidate what class(es) of proteases are prevalent in AIP-treated biofilms, the effluent from a detaching biofilm was assayed for protease activity in the presence of protease inhibitors or activating agents. The addition of EGTA, an inhibitor of the metalloprotease aureolysin [16], had a negligible effect on overall protease activity (Figure 8C). The addition of PMSF, a potent serine protease inhibitor, however reduced overall protease activity to almost undetectable levels. Lastly, the addition of DTT, a reducing agent used to activate thiol proteases [48], did not significantly change protease activity in the effluents. These results suggest that serine proteases are the dominant, detectable secreted protease in AIP-treated biofilms.

### Protease activity is required for biofilm detachment

**Figure 9:**
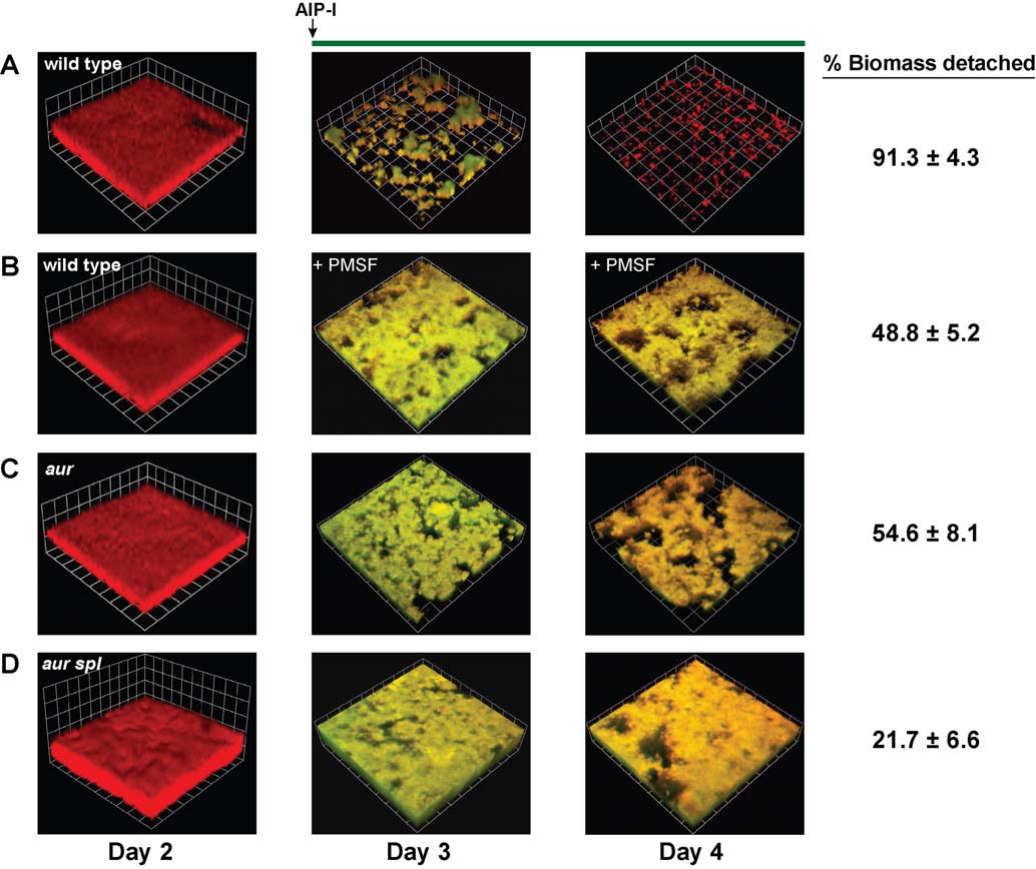
Effect of a serine protease inhibitor and protease deficient mutants on AIP-I mediated biofilm detachment. Columns show CLSM reconstructions of biofilms at day 2, day 3 and day 4. Biofilms were grown for 2 days and the growth media was supplemented with AIP-I or AIP-I+PMSF as indicated. Greenish yellow color indicates expression of the *agr* P_3_-GFP reporter, and the red color is from propidium iodide present in the growth medium. (A) Wild type biofilm (AH462) supplemented with 50 nM AIP-I. (B) Wild type biofilm (AH462) supplemented with 50 nM AIP-I and 10 µM PMSF. (C) Aureolysin (Δ*aur*) mutant biofilm (AH789) supplemented with 50 nM AIP-I. (D) Aureolysin Spl (Δ*aur* Δ*spl*) double mutant biofilm (AH788) supplemented with 50 nM AIP-I. CSLM reconstructions are representative of three separate experiments and each side of a grid square represents 20 µM. Percent biomass detached was calculated by COMSTAT analysis comparing biomass at day 2 to biomass at day 4.

**Figure 10:**
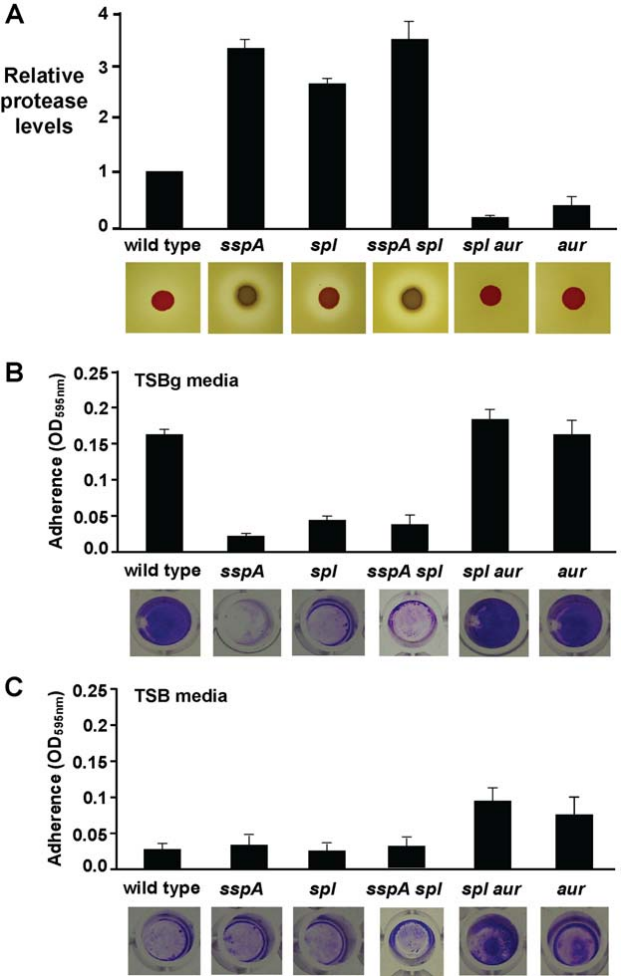
Extracellular protease activity and biofilm formation of protease mutants. (A) Relative protease levels detected in wild type and protease mutants grown in broth culture. Images show bacterial colonies and zones of clearing caused by protease activity on milk agar plates. (B–C) Biofilm formation of wild type and protease mutants in wells of microtiter plates. Graphs show quantitation of biofilm biomass attached to microtiter plate grown in either (B) TSBg or (C) TSB. Images below each graph are of crystal violet stained biofilms in wells of microtiter plates.

With our observation that serine proteases are abundant in detaching biofilms, we examined the effect of a serine protease inhibitor on AIP-mediated detachment. The addition of 10 µM PMSF in combination with AIP-I to an *S. aureus* biofilm significantly reduced the level of detachment compared with AIP-I alone (Figure 9A vs. B). However, 48.8% (±5.2) of the biomass still detached indicating that serine proteases are necessary but not sufficient for complete detachment. To further examine the mechanism, knock-out mutations were constructed in the genes encoding the V8 (SspA) and SplABCDEF serine proteases. Surprisingly, *sspA*::Tet and *Δspl*::Erm single mutants, and an *sspA*::Tet *Δspl*::Erm double mutant, all increased extracellular protease levels (Figure 10A) and eliminated biofilm formation under microtiter plate conditions (Figure 10B & 10C).

To block other extracellular proteases, a mutation was constructed in the gene encoding aureolysin (Aur). Aur is a metalloprotease that is required to initiate a zymogen activation cascade [49],[50], starting with the V8 protease [51], which in turn activates the SspB cysteine protease [52]. The activation mechanism of the ScpA cysteine protease remains unresolved [49]. In contrast to the serine protease mutations, introduction of the *Δaur* deletion into *S. aureus* reduced extracellular protease levels (Figure 10A) and did not affect biofilm formation (Figure 10B). Interestingly, under conditions of high *agr* activity, the *Δaur* deletion displayed improved biofilm formation versus wild-type (Figure 10C). In biofilm detachment tests, the *Δaur* mutant reduced AIP-mediated detachment, but 54.6% (±8.1) of the biomass still detached (Figure 9C). Considering the Spl proteases are not zymogens [53], we examined the combined effects of the Aur cascade and the Spl proteases by constructing an *Δaur Δspl*::Erm double mutant. The *Δaur Δspl* strain possessed very low levels of extracellular protease activity (Figure 10A) and had a minor enhancement in biofilm formation (Figure 10B). Similar to the *Δaur* mutant, the *Δaur Δspl* double mutant also displayed improved biofilm formation versus wild-type under conditions of high *agr* activity (Figure 10C). After AIP-I addition, only 21.7% (±6.6) of the *Δaur Δspl* mutant biomass detached in comparison to 91.3 (±4.3) of the wild-type strain (Figure 9D). These experiments indicate that the extracellular proteases have anti-biofilm properties and they demonstrate that *agr*-mediated biofilm detachment requires the activity of these proteases.

## Discussion

The majority of studies on biofilm detachment have focused on factors capable of initiating the process, such as nutrient availability [54],[55], nitric oxide exposure [56], oxygen tension [57], iron salts [58], chelators [59], and signaling molecules [60]–[63]. Alternatively, detachment studies have addressed effector gene products that contribute to the dissolution of the biofilm, including surfactants [10],[13],[64],[65], hydrolases [66],[67], proteases [37]–[39], and DNase [68]. Here were do both, by demonstrating that the increasing AIP levels or lowering available glucose can function as a *S. aureus* biofilm detachment signal by activating the *agr* quorum-sensing system, resulting in increased levels of extracellular proteases needed for the detachment mechanism. Importantly, *agr*-mediated detachment also restores antibiotic sensitivity to the released bacteria, suggesting the mechanism could be a target for treating biofilm infections.

These results are in accord with previous studies showing that *agr* mutants have a propensity to form biofilms [13],[14] and that cells actively expressing *agr* leave biofilms at a high frequency [19]. Our findings also explain why *S. aureus* biofilm formation requires glucose supplementation to growth media. Unless the *agr* system is repressed or inactivated, or the enzymes mediating detachment are inhibited, *S. aureus* will remain in a planktonic state. The presence of glucose is known to represses RNAIII through a nonmaintained pH decrease to ∼5.5 [20], resulting from the secretion of acidic metabolites. The RNAIII repression is not due to glucose itself, but results from the mild acid conditions [26] and can be mimicked with other carbon sources, such as galactose [20], that also lower the media pH. In microtiter biofilm experiments, we found these alternative pH-lowering carbon sources could substitute for glucose in facilitating biofilm formation (data not shown). The molecular mechanism through which low pH inhibits RNAIII expression remains to be determined. In the host, many niches colonized by *S. aureus* are maintained in lower pH ranges, such as the skin and vaginal tract [26], colonization sites that repress *agr* function could promote biofilm formation.

Based on our findings, we propose that the *S. aureus agr* quorum-sensing system controls the switch between planktonic and biofilm lifestyles. When the *agr* system is repressed, cells have a propensity to attach to surfaces and form biofilms as detachment factors are produced at low levels. In our detachment model, dispersal of cells from an established biofilm requires reactivation of the *agr* system and occurs through a protease-mediated, *ica*-independent mechanism. Yarwood et al. demonstrated through time-course, flow cell studies that reactivation of *agr* does occur in a biofilm [19], presumably through autonomous AIP production that reaches local concentrations high enough to activate *agr*. Under these fixed conditions, the *agr* system may function primarily as a mechanism to detach clumps (also called emboli) that seed new colonization sites.

In the experiments presented herein, we have employed growth conditions that tip the balance of the *agr* system, allowing an investigation into full *agr* reactivation within an established biofilm. This delicate balance can be offset with an increase in local AIP concentration or through changing environmental conditions, both situations that induce *agr* and result in massive dispersion of the cells. Biofilms are dynamic and dispersal is always operating [11], but accelerated detachment has been observed in response to changing environmental conditions, such as oxygen levels [57],[69], nutrient depletion [54], changing nutrient composition [55], or increased concentration of quorum-sensing signals [61]. An *S. aureus* biofilm growing *in vivo* is likely to encounter a changing physiochemical environment, which could serve as a cue to induce accelerated detachment through an *agr*-mediated mechanism.

*S. aureus* has been reported to form biofilms through an *ica*-dependent mechanism suggesting that PIA could have a role in detachment [34],[36]. We observed no defect in microtiter or flow cell biofilm formation using an *ica* mutant of SH1000 (Figure 7). Our findings support the growing evidence that PIA is not a major matrix component of *S. aureus* biofilms, as exogenous addition of dispersin B, an N-acetyl-glucosaminidase capable of degrading PIA, has little effect on established biofilms of SH1000 and other *S. aureus* strains [70]. In contrast, dispersin B does detach *S. epidermidis* biofilms indicating a more significant role for PIA in the *S. epidermidis* matrix structure [70]. Our experiments with proteinase K and the *S. aureus* proteases indicate that some proteinaceous material is important for SH1000 biofilm integrity, and this result supports a number of recent studies demonstrating that proteases can inhibit biofilm formation or detach established biofilms from many *S. aureus* strains [35], [37]–[39]. It is not clear whether *agr*-mediated detachment will function in *S. aureus* strains that produce an *ica*-dependent biofilm.

In this study, we document a role for the Aur and Spl proteases in biofilm detachment. Global expression analysis has shown that activation of the *agr* quorum-sensing system results in up-regulation of extracellular proteases (Aur, SplABCDEF, ScpA, SspAB) and down-regulation of many surface proteins [45],[46]. However, the target of these *agr* controlled proteases is not clear. One potential target is the surface adhesins, and possible candidates include the surface proteins Atl, Bap, and SasG, all of which have reported roles in biofilm formation [41], [42], [71]–[73]. Atl is additionally known to require proteolytic processing for activation, and this processing is PMSF inhibited [74]. Other possibilities include microbial surface components recognizing adhesive matrix molecules (MSCRAMMs), which are important for adherence to the extracellular matrices of mammalian cells [40]. Also, the *S. aureus* secreted proteases are known to activate lipase (Sal-1 and Sal-2) precursors [75] and process other secreted enzymes, such as staphylococcal nuclease [76],[77].

In addition to proteases, there may be other *agr* regulated factors that contribute to biofilm detachment. Surfactant-like molecules, such as δ-toxin, are induced by the *agr* system and may exert dispersal effects on biofilms [13],[78]. There is growing evidence that extracellular DNA (eDNA) is an important *S. aureus* biofilm matrix component [24],[70], and expression of staphylococcal nuclease is reported to be under control of the *agr* system [18]. Thus, while *agr* induced proteases are required for the detachment phenotype, the *agr* controlled expression of an array of factors (proteases, nuclease, surfactants) may also contribute to the biofilm detachment mechanism.

There is increasing interest in understanding how bacteria detach from biofilms and initiate colonization of new surfaces. The regulation of quorum-sensing systems may be one mechanism by which many bacteria control biofilm formation and dispersal. Quorum-sensing has been implicated in dispersal of biofilms formed by *Yersinia pseudotuberculosis* [79], *Rhodobacter sphaeroides* [80], *Pseudomonas aureofaciens* [81], *Xanthomonas capmestris* [62], and *Serratia marceascens* [61]. However, homoserine lactone signals play a divergent role in *Pseudomonas aeuruginosa* [12], *Pseudomonas fluorescens* [82], and *Burkholderia cepacia* [83], where the active versions of these quorum-sensing systems are necessary for biofilm formation and robustness under some growth conditions. In both cases, it appears quorum-sensing plays a significant role in biofilm development and determining the environmental stimuli that modulate quorum-sensing activity will provide insight on bacterial colonization, detachment, and dispersal to new sites.

## Materials and Methods

### Strains and growth conditions

The bacterial strains and plasmids used in this study are described in Table 1. *S. aureus* or *Escherichia coli* were grown in tryptic soy broth (TSB) or on tryptic soy agar (TSA) with the appropriate antibiotics for plasmid selection or maintenance (erythromycin 10 µg/ml; chloramphenicol 10 ug/ml; tetracycline 5 ug/ml) and incubated at 37°C. Plasmid DNA was prepared from *E. coli* and transformed by electroporation into *S. aureus* RN4220 as described [84]. Plasmids were moved from RN4220 into other *S. aureus* strains by transduction with bacteriophage α80 [85] or by purifying the plasmid DNA and transformed by electroporation into appropriate strains. To move *sspA* and *splABCDEF* mutations into appropriate genetic backgrounds, phage transduction with α80 was used as described [85]. To construct the Δ*aur* mutation, the pKOR1-*aur* plasmid was used as described [16]. Fluorescence measurements with *S. aureus* strains containing pDB59 were performed as previously described [27].

**Table 1:** Strain and plasmid list

Strain or plasmid	Genotype	Resistance	Source or reference
*Escherichia coli*			
DH5α-E	Cloning strain	None	Invitrogen
AH394	ER2566/Δ*gshA::cat*	Cam	[27]
AH426	AH394/pDnaB8–AIPI	Amp	[27]
AH495	AH394/pDnaB8–AIPII	Amp	[27]
AH496	AH394/pDnaB8–AIPIII	Amp	[27]
*Staphylococcus aureus*			
RN4220	Restriction mutant of 8325-4	None	[85]
SH1000	*rsbU* positive derivative of 8325-4, *agr* Type I	None	[89]
SH1001	SH1000/*Δagr::tet*	Tet	[89]
FRI1169	*agr* Type I	None	[31]
SA502A	*agr* Type II	None	[15]
ATCC25923	*agr* Type III	None	ATCC
KB600	Δ*spl::erm*	Erm	[90]
SP6391	*sspA::erm*	Erm	[50]
DU1126	*sspA::tet*	Tet	[91]
MN8	Δ*ica::tet*	Tet	[92]
AH462	SH1000/pDB59	Cam	[16]
AH500	SH1000/pAH9	Erm	This work
AH595	SH1000/Δ*ica::tet*	Tet	This work
AH596	SH1000/pDB59+pAH9	Cam, Erm	This work
AH703	SH1000/Δ*aur*	None	This work
AH741	SH1000/*sspA::erm*	Erm	This work
AH751	SH1000/Δ*spl::erm*	Erm	This work
AH750	SH1000/Δ*aur* Δ*spl*::*erm*	Erm	This work
AH788	AH750/pDB59	Cam, Erm	This work
AH789	AH703/pDB59	Cam	This work
AH860	SH1000/Δ*spl::erm sspA::tet*	Erm, Tet	This work
AH861	SH1001/pDB59+pAH9	Cam, Erm	This work
Plasmids			
pDB59	P_3_-GFP reporter	Amp, Cam	[19]
pAH9	*sarA* promoter P_1_-RFP	Amp, Erm	This work
pDNAB8-AIPI	AIP-I intein plasmid	Amp	[27]
pDNAB8-AIPII	AIP-II intein plasmid	Amp	[27]
pDNAB8-AIPIII	AIP-III intein plasmid	Amp	[27]
pKOR1-*aur*	*aur* knockout vector	Amp, Cam	[16]

### Construction of an RFP reporter plasmid

The *sarA* P1 promoter region was PCR amplified from SH1000 genomic DNA with oligonucleotides (for 5′-GTTGTT*AAGCTT*CTGATATTTTTGACTAAACCAAATGC-3′, rev 5′-GTT*GGATCC*GATGCATCTTGCTCGATACATTTG-3′), digested with HindIII and BamHI, and cloned into the erythromycin shuttle plasmid pCE107 [19]. The mCherry (RFP) gene was PCR amplified from pRSET-mCherry [86] with oligonucleotides incorporating a 5′ ribosome binding site and KpnI site and a 3′ EcoRI site (for 5′-GTT*GGTACC*TAGGGAGGTTTTAAACATGGTGAGCAAGGGCGAGGAGG-3′, rev 5′-GTT*GAATTC*TTACTTGTACAGCTCGTCCATGCC-3′). The mCherry fragment was cut with KpnI and EcoRI and cloned downstream of the *sarA* promoter to generate a constitutive RFP expressing plasmid called pAH9.

### Monitoring protease activity

Milk agar plates for detection of protease activity consisted of 3 g/L Tryptic Soy broth, 20 g/L non-fat dry milk, and 15 g/L agar. To determine relative protease activities of strains, assays were performed as described previously using the Azocoll (Calbiochem) reagent [48]. For measuring protease levels in biofilm effluents, 100 mL of effluent was collected on ice (∼12 hours) after AIP addition to the biofilm medium. Cells were removed from the effluents through centrifugation and filtering, and ammonium sulfate was added to 60% over one hour at 4°C to concentrate proteins. The precipitated proteins were pelleted by centrifugation at 19,000 rpm for 30 min, and the pellet was washed and resuspended in 1 ml with 10 mM Tris pH 7.5. For the protease assay, the reaction mixture was supplemented with either 1 mM EGTA, 200 µM PMSF, or 1 mM DTT to gauge relative levels of protease classes.

### Biofilm experiments

Microtiter plate biofilms were performed as described [87] except that the plates were incubated at 37°C with shaking at 200 rpm for 12 hours. For flow cell experiments, AIPs were generated using the DnaB intein method, and the AIP concentrations were determined as previously described [27]. AIPs stocks (∼20 µM) were stored in 100 mM phosphate [pH 7], 50 mM NaCl, 1 mM tris(2-carboxyethyl) phosphine (TCEP) and were diluted into the biofilm flow medium to a final concentration of 50 nM. When required, 5 µg/ml of erythromycin and/or chloramphenicol were added to the flow cell media to maintain plasmids. The growth medium for flow cell biofilms consisted of 2% TSB plus 0.2% glucose unless otherwise indicated. Flow cell biofilm experiments and confocal microscopy were performed as previously described [19]. Flow cells were inoculated with overnight cultures diluted 1:100 in sterile water and laminar flow (170 µl/min) was initiated after one hour incubation. Confocal microscopy was performed using a Radiance 2100 system (Biorad) with a Nikon Eclipse E600 microscope. Confocal images were processed using Velocity software (Improvision, Lexington, Mass.). Biofilm biomass was quantified with the COMSTAT program [88] and percent biomass detached was calculated by subtracting biomass present at day 4 from day 2. To quantitate the number of bacteria detaching from a biofilm, 1 ml of flow cell effluent was collected on ice at indicated time points. The collected effluent was vortexed and sonicated in a water bath for 10 minutes to break up clumps, and serial dilutions were plated on TSA plates to determine colony forming units (CFUs). For the Proteinase K detachment experiments, the enzyme (Sigma-Aldrich) was suspended in water and added to the media reservoir at a final concentration of 2 µg/ml.

### Antibiotic sensitivity

*S. aureus* biofilms were grown for two days in a flow chamber lined with removable polycarbonate coupons (Flow Cell FC271, Biosurface Technologies, Bozeman MT). Biofilm effluents were collected on ice ∼24 hours after AIP-I addition. In parallel, coupons with biofilm growth were removed from flow cells not exposed to AIP-I. Both detached bacteria and the biofilms were exposed to the indicated levels of rifampicin for six hours. Subsequently, cells were vortexed, and the coupons were sonicated in a water bath to break up the biofilm or cell clumps. Serial dilutions were plated on TSA to determine surviving CFU's.

## Supporting Information

Video S1 CLSM time course of AIP mediated detachment.
(0.95 MB AVI)
